# Cellular and molecular mechanism study of declined intestinal transit function in the cholesterol gallstone formation process of the guinea pig

**DOI:** 10.3892/etm.2014.1943

**Published:** 2014-09-01

**Authors:** YING FAN, SHUODONG WU, ZHENHUA YIN, BEI-BEI FU

**Affiliations:** Department of the Second General Surgery, Sheng Jing Hospital of China Medical University, Shenyang, Liaoning 110004, P.R. China

**Keywords:** cholesterol gallstone, intestinal transit, interstitial cells of Cajal, c-kit, stem cell factor

## Abstract

The aim of this study was to investigate the cellular and molecular mechanisms of declined intestinal transit (IT) function in the cholesterol gallstone (CG) formation process. Forty guinea pigs were divided into an experimental group (EG) and a control group (CoG), and the reverse transcription-polymerase chain reaction (RT-PCR) was performed for the analysis of c-kit and stem cell factor (scf) mRNA expression in the small bowel. In addition, immunofluorescence staining and confocal laser microscopy were performed for the observation of the changes in the number of interstitial cells of Cajal (ICCs) in the terminal ileum of each group. RT-PCR showed that, compared with the CoG, the intestinal c-kit and scf mRNA expression levels in the EG were significantly decreased; the average positive area of ICCs in the ileum in the EG was also significantly reduced. During the diet-induced CG formation procedure, the c-kit and scf mRNA expression levels in the small intestine decreased and the number of ICCs decreased. Inhibition of the c-kit/scf pathway may be involved in the declined IT function during the CG formation process.

## Introduction

The cause of cholesterol gallstone (CG) formation is complicated; however, the role of small intestine motility has been gradually noted (1,2). In our previous study, it was found that the intestinal transit (IT) function was decreased in the diet-induced Golden hamster CG model (3), indicating that declined IT may play an important role in the process of CG formation.

It has been demonstrated that the interstitial cells of Cajal (ICCs) are the pacemaker cells of the gastrointestinal slow wave, regulating the pacing and spreading of smooth muscle activity and participating in the conduction of nerve signal pathways (4–6). ICCs can express the c-kit proto-oncogene, which encodes a tyrosine kinase membrane receptor. The signaling pathway resulting from the combination of c-kit and its ligand stem cell factor (scf) plays an important role in the development, differentiation and phenotypic maintenance of ICCs (7,8). However, whether the declined IT in the high-cholesterol diet-induced guinea pig CG model is caused by abnormalities in the ICCs and c-kit/scf pathway remains to be investigated. In this experiment, immunofluorescence staining and the reverse transcription-polymerase chain reaction (RT-PCR) were performed to detect the number of ICCs in guinea pig small intestine tissue and to analyze the mRNA expression of c-kit and scf, respectively. The aim of the study was to explore the cellular and molecular mechanisms of the IT decline during the CG formation process of the guinea pig.

## Materials and methods

### Animal model and grouping

The experiment was approved by the Ethics Committee of Sheng Jing Hospital of China Medical University (Shenyang, China). Forty healthy male guinea pigs, aged four weeks and weighing 120–125 g, were provided by the Experimental Animal Center of Sheng Jing Hospital of China Medical University. The animal license number was SCXK (Liao) 2009–0016. The animals were randomly divided into two groups: The experimental group (EG, n=20) and the control group (CoG, n=20). The animals in the EG were fed a high-cholesterol diet (2% cholesterol, purchased from Sinopharm Chemical Reagent Co., Ltd., Shanghai, China), while those in the CoG received a normal diet. Each cage was limited to two animals. The experiment was performed under standard laboratory conditions (12-h light/dark cycle, 21–24°C, humidity at 50–55%). Free feeding with cabbage every two days was maintained for eight weeks prior to the experiment.

### Bile smear observation by polarizing microscopy and infrared spectrum analysis of the biliary calculus**.**

Bile was obtained from the gallbladder with the fine-needle penetration method. A small amount of bile was dropped onto the slide to form the bile smear, and a DP-71 polarized light microscope (Olympus, Tokyo, Japan) was used to observe the cholesterol crystals. The biliary calculus was collected following a cholecystectomy, washed with distilled water and dried naturally, prior to a 2-mg portion being mixed with 100 mg KBr. The stone and KBr were fully milled for 10 min and baked under an infrared lamp for ~30 min prior to being pressed into a transparent sheet with a 3000X tablet machine (BSTD Co, Ltd., Tianjin, China). An FT-IR-55 infrared spectrometer (Bioon Co., Ltd, Bern, Switzerland) was used to analyze the composition of the sheet.

### Observation of the guinea pig small intestine with immunofluorescence staining and laser confocal microscopy

The guinea pigs were anesthetized, and ~2 cm terminal ileum was reserved. The intestinal contents were then cleaned with 0.9% sodium chloride solution. One end of the terminal ileum was ligated, and acetone was injected through the reserved end of the intestine. Subsequent to the segment fully expanding, the reserved end was ligated and the intestine was retained in a refrigerator at 4°C. The intestine was cut along the longitudinal axis with microsurgical scissors, and then cut into pieces measuring 3×3 mm. Under the operating microscope, the intestinal mucosa and submucosa were peeled away with microdissection forceps, while the muscular layer was reserved. The muscular layer was then stretched and rinsed with 0.01 mol/l phosphate-buffered saline (PBS), prior to being treated with 0.03 mol/l PBS-Tween 20 for 1 h and sealed with normal goat serum for 1 h. Diluted rabbit anti-mouse c-kit (ACK II) primary antibody (Beijing Biosynthesis Biotechnology Co., Ltd., Beijing, China) was subsequently added at a dilution of 1:100 followed by incubation overnight. On the next day, 1:100 cyanine 3 (CY3)-labeled goat anti-rabbit secondary antibody (Beijing Biosynthesis Biotechnology Co., Ltd.) was added and incubated in the dark for 1 h. DAPI was added for the re-staining, and the slide was then mounted with glycerol mounting medium and the coverslip. The c-kit-positive ICCs were counted and images were captured using the Eclipse IZ laser confocal microscope (Nikon Corp., Tokyo, Japan). The percentage ICC-positive area was automatically calculated with the image analysis software of the Nikon Eclipse IZ system.

### Extraction of total RNA

Fresh intestinal tissue (200 mg) of the guinea pig was placed in liquid nitrogen, then repeatedly crushed prior to the addition of 1 ml TRIzol^®^ solution (Invitrogen Life Technologies, Carlsbad, CA, USA). The total RNA was extracted according to the TRIzol reagent kit instructions (Invitrogen Life Technologies).

### c-kit and scf gene cloning with RT-PCR

The purified RNA was used to synthesize cDNA through the RT reaction. According to the RT kit (Takara Corporation, Dalian, China), the first chain of cDNA was initially synthesized and used as the template for the PCR. The gene primer sequences were as follows: c-kit, upstream 5′-CACAGAGGCTTAGCGG-3′, and downstream 5′-CGTGAAGGCAACATACC-3′; scf, upstream 5′-GCAGCATAATACCACG-3′, and downstream 5′-AATACCATCATCCGTTC-3′; internal reference GAPDH, upstream 5′-ACCACAGTCCATGCCATCAC-3′, and downstream 5′-TCCACCACCCTGTTGGGTA-3′. The PCR products were then separated by electrophoresis, with reference to the blank control and molecular size of the DNA Ladder Marker. The positive electrophoretic bands were confirmed and the desired strips were obtained for the recovery of the c-kit and scf DNA fragments according to the instructions of the gel extraction kit (Takara Corporation). Following the 1.5% agarose gel electrophoresis, the PCR products were stained with ethidium bromide, observed and photographed under ultraviolet light, with ΦX174/*Hae*III and DL2000 DNA Marker as the molecular weight standards. A GIS-2020 digital gel-scanning imaging and analysis system (Dobio Co., Ltd., Shanghai, China) was used to perform the electrophoretic band analysis, and the mRNA expression levels of c-kit and scf were calculated according to the relative expression levels of the internal reference mRNA GAPDH.

### Statistical analysis

Statistical analyses were performed using SPSS 11.5 statistical software (SPSS, Inc., Chicago, IL, USA), and all experimental data are expressed as the mean ± standard deviation. Inter-group comparisons were conducted with the Student’s t-test, and the rate comparisons were performed with the χ^2^ test. P<0.05 was considered to indicate a statistically significant difference.

## Results

### Guinea pig growth and stone formation

Three animals in the EG died, with the mortality rate at 15%. In the later stage of the feeding time, the animals moved slowly, exhibited dull responses, ate less and shed hair. The autopsies revealed that the causes of death were pneumonia (n=1), splenic abscess (n=1) and cervical lymphadenitis (n=1). There were no cases of mortality in the CoG, and the guinea pigs grew in good condition.

At the end of the lithogenic period, the dissection revealed that the gallbladders of the EG guinea pigs were swollen, with evident granular, yellow, single or multiple stones. By contrast, the gallbladders of the CoG were normal with clear and bright bile. There was no stone formation in the CoG guinea pigs, and the stone formation rate was 0% (0/20); in the EG, stones formed in the remaining 17 guinea pigs, and the stone formation rate was 100% (17/17). Polarized light microscopy revealed no cholesterol crystals in the gallbladder bile of the CoG guinea pigs, while the typical needle-like crystals of cholesterol could be observed in the EG. Infrared spectroscopy of the stone-KBr sheets showed that at the wavelengths of 1,418 cm^−1^ and 1,640 cm^−1^, clear cholesterol absorption peaks could be observed.

### Small intestine c-kit and scf mRNA expression results

**Figure 1:**
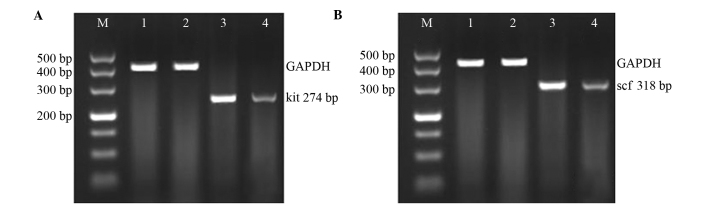
Gel electrophoresis results of (A) c-kit and (B) stem cell factor cDNA by the reverse transcription-polymerase chain reaction. Lanes: M, DNA ladder marker; 1 and 3, control group; 2 and 4, experiment group.

Compared with the CoG, the small intestinal mRNA expression levels of c-kit (0.316±0.056 vs. 0.912±0.103; t=6.582, P<0.01) and scf (0.499±0.012 vs. 0.899±0.124; t=6.163, P<0.01) in the EG guinea pigs were significantly decreased (Fig. 1A and B).

### Immunofluorescence staining results of the guinea pig small intestine

**Figure 2:**
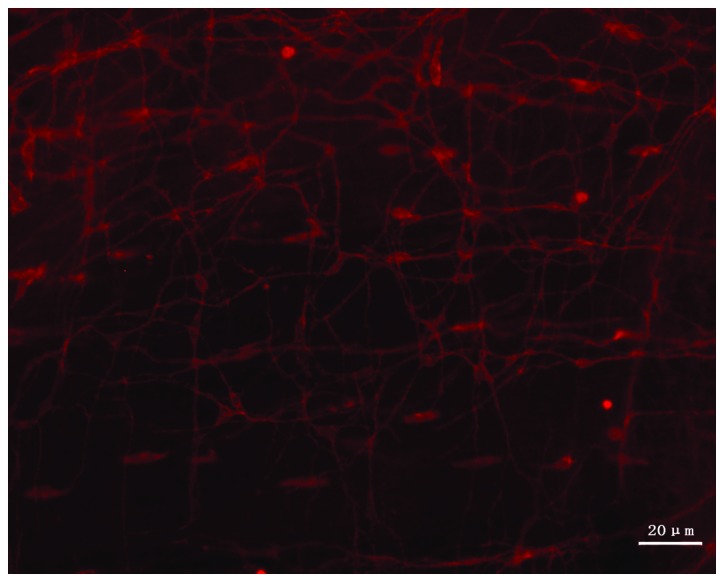
c-kit-positive interstitial cells of Cajal of the small intestine in the control group (confocal microscope c-kit immunofluorescence stain; magnification, ×200).

**Figure 3:**
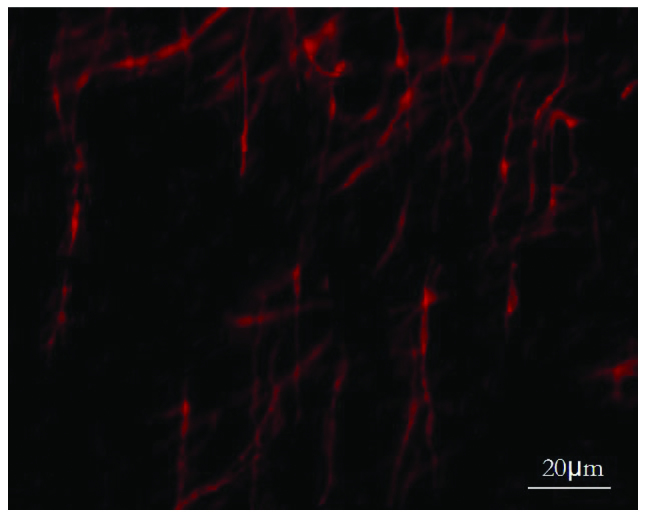
c-kit-positive interstitial cells of Cajal of the small intestine in the experimental group (confocal microscope c-kit immunofluorescence stain; magnification, ×200).

The c-kit-positive ICCs were present in the muscular layer of the small intestine in the guinea pigs. When stained by CY3-conjugated secondary antibody, the cells exhibited red fluorescence with confocal laser microscopy, and this was significantly highlighted against the black background. Under the confocal microscope, ICCs were observed to exhibit a fusiform or satellite shape, with large oval DAPI-stained (blue) nuclei, little perinuclear cytoplasm and between two and five long, branched cell processes. The cells thus appeared to be spindle- or satellite-like cells. The ICCs were connected to each other, forming a network-like structure (Fig. 2). The small intestinal slides of the EG also exhibited c-kit-positive ICCs, showing no significant difference in single cell morphology with the CoG under the light microscope, while the number of ICCs observed within the vision field was significantly reduced in the EG, and the network structure was found to have disappeared (Fig. 3).

For the comparative observation of the slides of the CoG and EG, three fields of each slide were randomly scanned, and the immunofluorescence image analysis software was used to count the percentage ICC-positive area. The results showed that the average ICC-positive area in the EG was significantly reduced when compared with that in the CoG (22.26±1.14 vs. 56.24±2.68%; t=15.256, P<0.01).

## Discussion

The incidence of CGs increases with age*,* and the condition exhibits a complex pathogenesis. In the past two decades, studies on the motility function of the small intestine-gallbladder have increased, showing that patients with CGs experience a decline in IT function, leading to a prolongation of the transit time of bile in the small intestine and increased levels of bile deoxycholic acid (DCA) (9,10). DCA is positively correlated with the cholesterol saturation index and cholesterol crystallization rate (11); furthermore, the gallbladder contraction and IT function during the digestion period are associated with the serum motilin level. The decline in IT induces disordered gallbladder emptying, and increases in bile concentration (12). Extension of the IT period also prolongs the enterohepatic circulation of bile acids, thus slowing the bile salt reabsorption rate. As a result, the bile salt content falls, which promotes cholesterol supersaturation and crystallization (13). However, the mechanism underlying the decline in IT has not been reported, to date.

In 1893, Cajal, the Spanish neuroanatomist, first reported the existence of a class of mesenchymal cells that were present in the gastrointestinal tract and exhibited a network structure, namely the ICCs (14). Recent studies have confirmed that ICCs play an important role in the pacing and dissemination of the smooth muscle activity in the gastrointestinal tract, and also in the nerve signal transduction pathway (15–18). ICCs can specifically express c-kit, a type III tyrosine kinase membrane receptor. In the present study, c-kit immunofluorescence staining was successfully performed on the whole layer slice of the small intestine of the guinea pig. The results showed that, during the CG formation process of the high-cholesterol diet-induced guinea pig, the number of intestinal ICCs was significantly decreased when compared with that of the CoG, and the network-like structure between the cells disappeared. This indicated that the changes in the number and function of ICCs and pacemaker cells in IT played an important role in the declined IT function during the process of stone formation.

It has also been indicated that the c-kit receptor has an important role in the development, growth and phenotypic maintenance of ICCs. Animals with a spontaneous c-kit mutation do not exhibit a normal occurrence and development of ICCs (19). Furthermore, when the c-kit neutralizing antibody ACK II was intraperitoneally injected into a new-born mouse, it was revealed that the normal contraction phase of the mouse intestine was disordered, and the electric slow wave activity and ICCs disappeared (20). Numerous studies have found a missing or damaged ICCs network in a variety of human diseases, including intestinal pseudo-obstruction, Hischsprung’s disease, Crohn’s disease, extensive bowel resection, hereditary transthyretin amyloidosis, diabetic gastroparesis and slow transit constipation (21–23). The experimental data from the present study showed that, in the small intestine of the high-cholesterol diet-induced CG guinea pig, the expression of c-kit mRNA was significantly decreased. This indicated that cholesterol could inhibit the expression of c-kit mRNA in intestinal tissues, which may explain the phenomenon of the reduced number of ICCs in the small intestine during the process of CG formation.

scf is a multifunctional growth factor involved in the growth regulation of a variety of cells in the body. scf can combine with its natural ligand, c-kit, forming the c-kit/scf system, which is associated with the differentiation, development, proliferation and phenotypic maintenance of ICCs (24). A previous study demonstrated that when a non-lethal mutation of scf occurred, heterozygous mice (Sl/Sld) exhibited severely disordered scf synthesis, and were only capable of synthesizing and secreting a small amount of soluble scf. Ten days after the birth of the Sl/Sld mice, the intestinal myenteric ICCs (ICCs-MY) showed poor development, forming a loose and atypical network structure compared with normal mice, with fewer cytoplasmic mitochondria and without membrane foveola. The ICCs-MY were difficult to identify 20 days after birth (25). In the ICC cell culture environment, scf should be added to aid the growth, development and phenotypic maintenance of ICCs (26). The aforementioned evidence shows that scf is essential for the occurrence, development and phenotypic maintenance of ICCs. In addition, the present experimental data show that, in the small intestine of the high-cholesterol diet-induced CG guinea pig, the expression of scf mRNA is significantly decreased, and cholesterol can inhibit the expression of scf mRNA in intestinal tissues, affecting the regulation of the number and function of ICCs.

In conclusion, the results of the present study indicated that the high-cholesterol diet was the causative factor that induced CG formation in the guinea pig. This occurred through the inhibition of the c-kit/scf signaling pathway, which resulted in a reduced number of ICCs and dysfunction in the guinea pig small intestine, including dysfunctional spontaneous rhythmic contraction, as well as the decline in IT function. This ultimately led to CG formation.
